# Fistula of acromioclavicular cyst treated with a staged reverse total shoulder arthroplasty: a case report

**DOI:** 10.1186/s12891-022-05976-5

**Published:** 2022-11-22

**Authors:** Mohammed Emam, Neil Singhani, Christine Persaud, William Aibinder

**Affiliations:** 1 Department of Physical Medicine and Rehabilitation, The Johns Hopkins University, 600 N. Wolfe St, Baltimore, MD 21287 USA; 2 Department of Orthopaedics and Rehabilitation Medicine, SUNY Downstate Medical Center, Brooklyn, NY USA; 3 Department of Orthopaedic Surgery, University of Michigan, Ann Arbor, USA

**Keywords:** Acromioclavicular cysts, Ultrasound, Cyst aspiration, AC joint fistula, Staged reverse total shoulder arthroplasty, Case report

## Abstract

**Background:**

Acromioclavicular (AC) joint cysts are relatively rare. There are two distinct etiologies of AC cysts. Type 1 is isolated to the AC joint, while type 2, is related to a tear or rupture of the rotator cuff (RC). The disease is usually a rare result of advanced AC joint arthritis or RC-tear arthropathy. Patients may present with signs and symptoms of RC impingement and tear. Conservative management may be used initially in asymptomatic individuals who are also not concerned with cosmesis. Aspiration and steroid injection of the cyst has been reported as one method of non-surgical management of these lesions, however, there is a high rate of recurrence.

**Case Presentation:**

We report a case of A 72-year-old right-handed female with past medical history of type two diabetes mellitus, chronic smoking, and a prior right RC repair with distal clavicle resection who presented with an AC joint cyst complicated by a draining fistula as a result of cyst aspiration and steroid injection. Due to the persistent drainage of the cyst and concern for infection, the patient was treated with a staged reverse shoulder arthroplasty given the setting of an irreparable rotator cuff tear and end-stage cuff-tear arthropathy.

**Conclusion:**

This case demonstrates an important complication of persistent draining fistula resulting from AC joint cyst aspiration and steroid injection in the setting of advanced RC-tear arthropathy. In immunocompromised patients, staged reverse shoulder arthroplasty should be considered for treatment of these draining fistulas especially when the concern for periprosthetic infection is high.

## Background

Acromioclavicular (AC) joint cysts are relatively rare [1]. There are two distinct etiologies of AC cysts. Type 1 is isolated to the AC joint, while type 2, is related to a tear or rupture of the rotator cuff (RC). The disease is usually a rare result of advanced AC joint arthritis or RC-tear arthropathy [2–4]. Patients may present with signs and symptoms of RC impingement and tear. Conservative management in the form of observation may be used initially in asymptomatic individuals who are also not concerned with cosmesis [2]. Aspiration and steroid injection of the cyst has been reported as one method of non-surgical management of these lesions, however, there is a high rate of recurrence [5].

## Case presentation

A 72-year-old right-handed female with past medical history of type two diabetes mellitus, chronic smoking, and a prior right rotator cuff (RC) repair with distal clavicle resection who presents with right shoulder swelling for four months. The swelling has progressively worsened and bothers her both symptomatically and due to cosmetic reasons. The swelling was accompanied by intermittent pain, 5/10 on numeric pain scale in the right shoulder. Her symptoms were aggravated by pushing/pulling actions as well as overhead movements. She denied antecedent trauma or injuries.

On physical examination, an approximately 2.5X2 cm non-tender, firm cyst overlying right acromioclavicular (AC) joint was observed. There was no erythema, drainage, or skin breakdown. Active range of motion included 90 degrees in abduction and flexion, otherwise normal internal and external rotation. There was no tenderness to palpation along acromion, AC joint, RC insertion, bicipital groove, or clavicle. Neer’s test and Hawkin’s test were positive. There was no scapular dyskinesia, or winging. There were no focal neurological deficits. Radial pulses were palpable and symmetric.

**Fig. 1:**
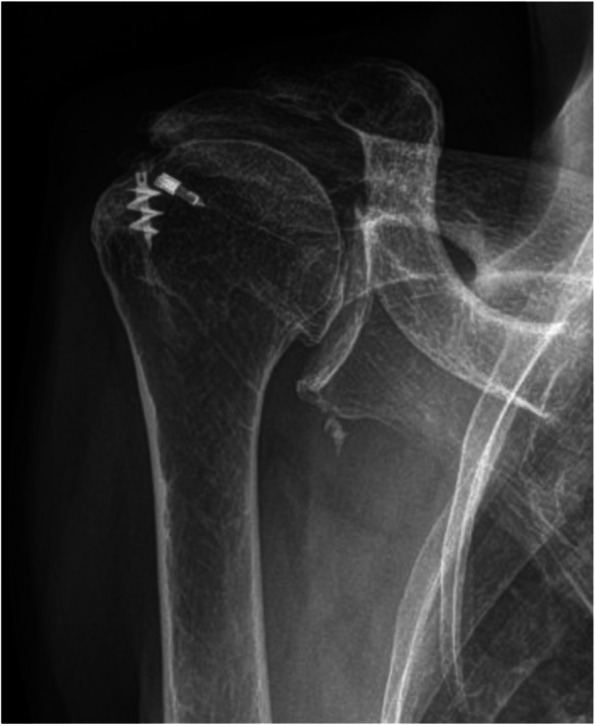
Preoperative grashey radiograph of the right shoulder demonstration superior migration of the humeral head with prior metallic anchor from a rotator cuff repair. The radiograph is consistent with rotator cuff tear arthropathy

**Fig. 2:**
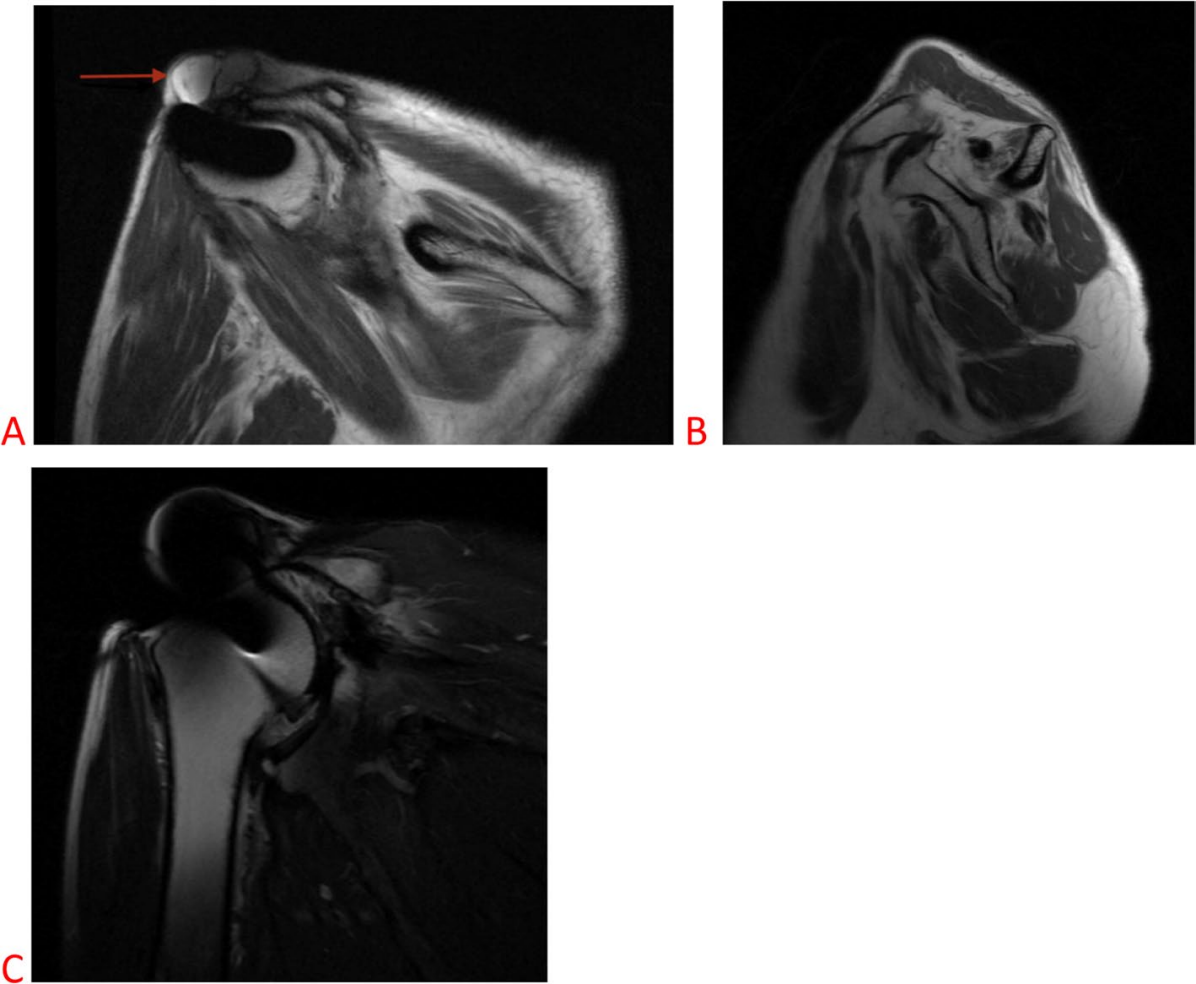
MRI of the right shoulder. **A** Coronal proton density image showing a cystic mass overlying the acromion (straight arrow); **B** Sagittal T1 MRI demonstrating severe fatty infiltration of the supraspinatus and infraspinatus muscles; and (**C**) Coronal T1 MRI demonstrating a massive rotator cuff tear with retraction of the superior rotator cuff to the level of the glenoid

A plain radiograph revealed a high-riding humerus with degenerative changes of the AC and glenohumeral (GH) joints (Fig. 1). A soft tissue mass was also observed overlying the acromion. A musculoskeletal ultrasound examination revealed a cyst located over and just lateral to the AC joint, atrophy and full thickness tear of the supraspinatus tendon. Magnetic resonance imaging (MRI) of her right shoulder without contrast revealed maceration of the glenoid labrum with homogenous fluid traversing through a retracted RC tear and distal clavicular resection superiorly to form a geyser phenomenon and an AC joint cyst (Fig. 2).

**Fig. 3:**
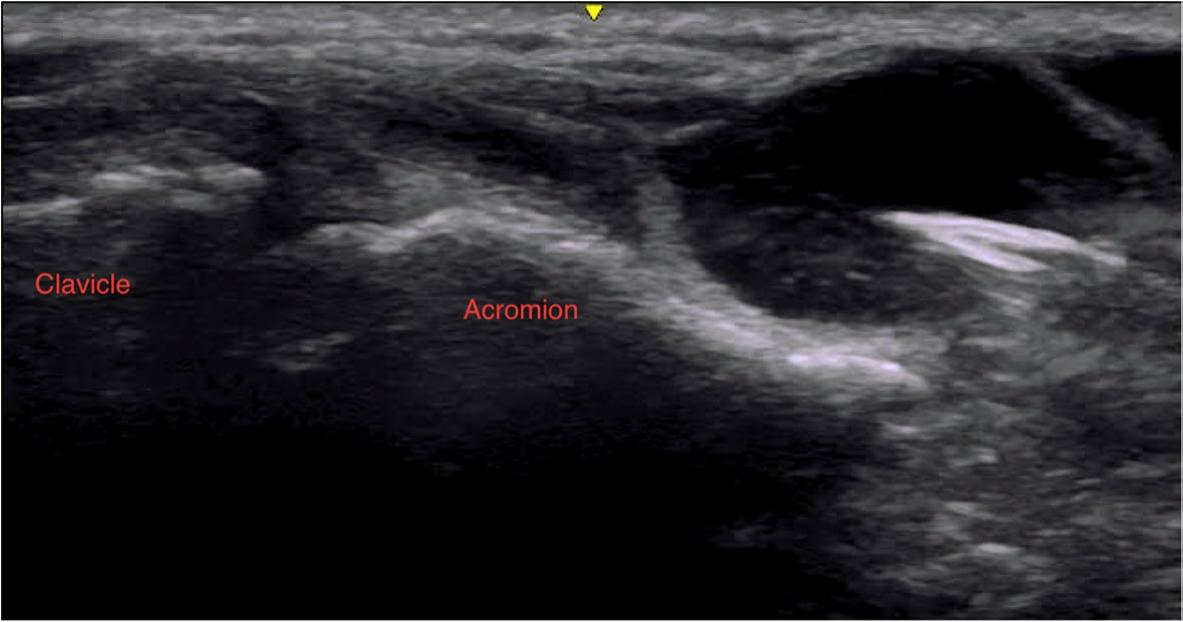
Ultrasound guided aspiration of the AC joint cyst extending from the AC joint capsule

The patient was referred for an ultrasound guided cyst aspiration (Fig. 3). After local skin anesthesia, an 18-gauge 1.5-inch needle was inserted under ultrasound guidance into the acromioclavicular cyst and 5 ml of clear viscous fluid was aspirated followed by injection with 1 ml of 40 mg methylprednisolone acetate and 1 ml of 1% lidocaine. The patient returned two weeks later with recurrence of the cystic swelling in the same location. A repeat aspiration 5 ml of clear viscous fluid under ultrasound guidance was performed. The aspirated fluid was tested for gram stain, culture, cell count, and crystals, all which came back normal.

**Fig. 4:**
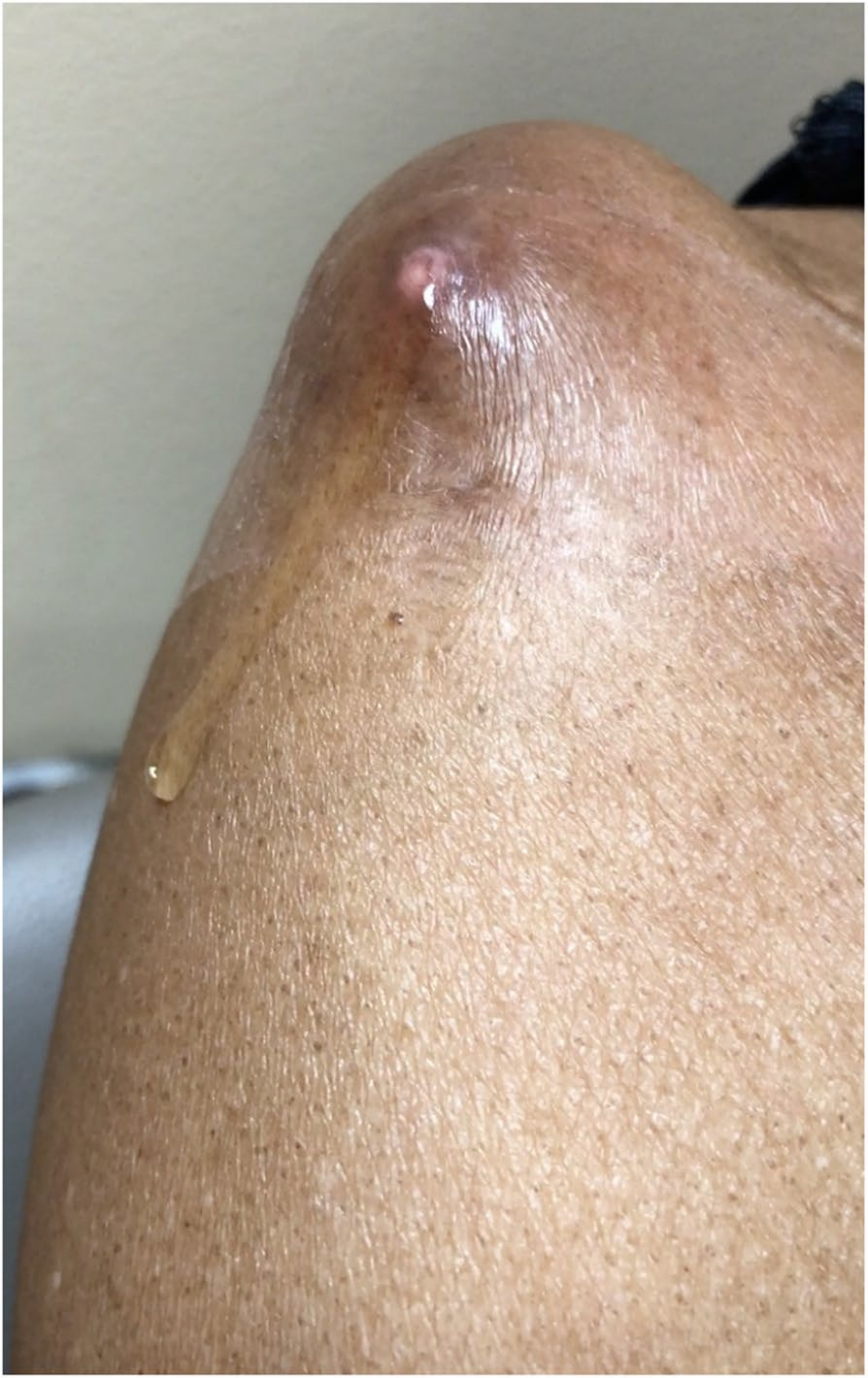
Preoperative image of right AC joint cyst with draining fistula

**Fig. 5:**
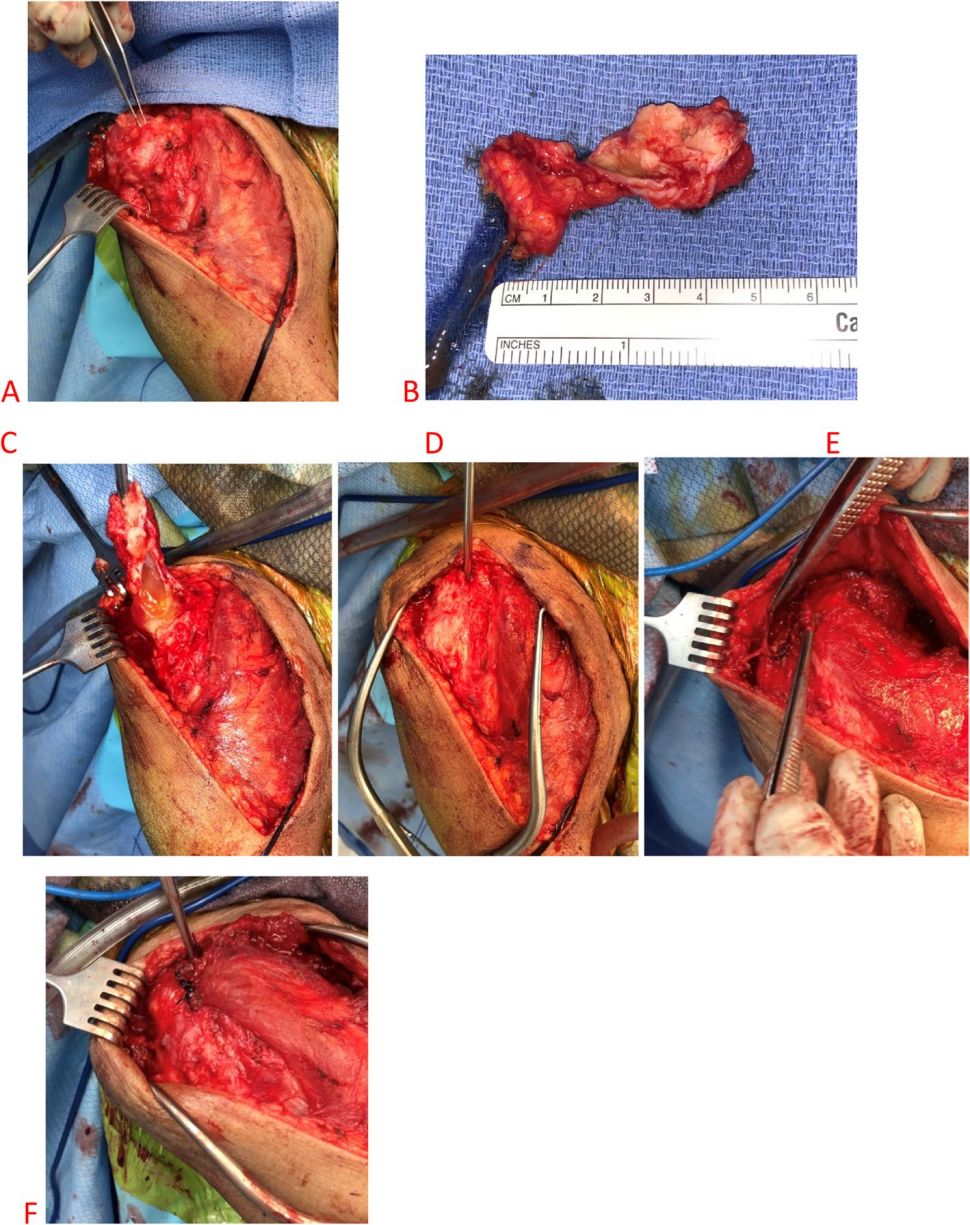
Intraoperative clinical images. **A** Cystic mass present arising from the AC joint (under the forceps); **B** Specimen of the cyst after excision; **C** Dissection of the cyst with rupture of the capsule after elevating off the AC joint demonstrating the cystic contents; **D** After cyst excision demonstration of an instrument through the AC joint with obvious communication into the intra-articular space; **E** Mobilization of the deltoid muscle (small forceps at the bottom of the image) and trapezius muscle (large forceps at top of image); **F** Closure and coverage of the AC joint after a pants-over-vest repair of the deltoid and trapezius muscles

**Fig. 6:**
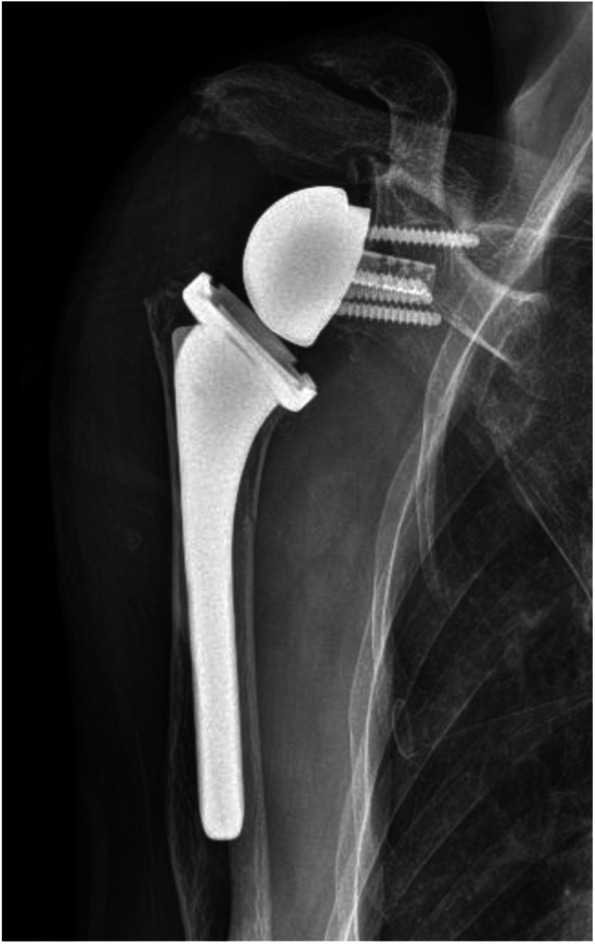
Postoperative anteroposterior radiograph of the right shoulder demonstrating a reverse total shoulder arthroplasty

**Fig. 7:**
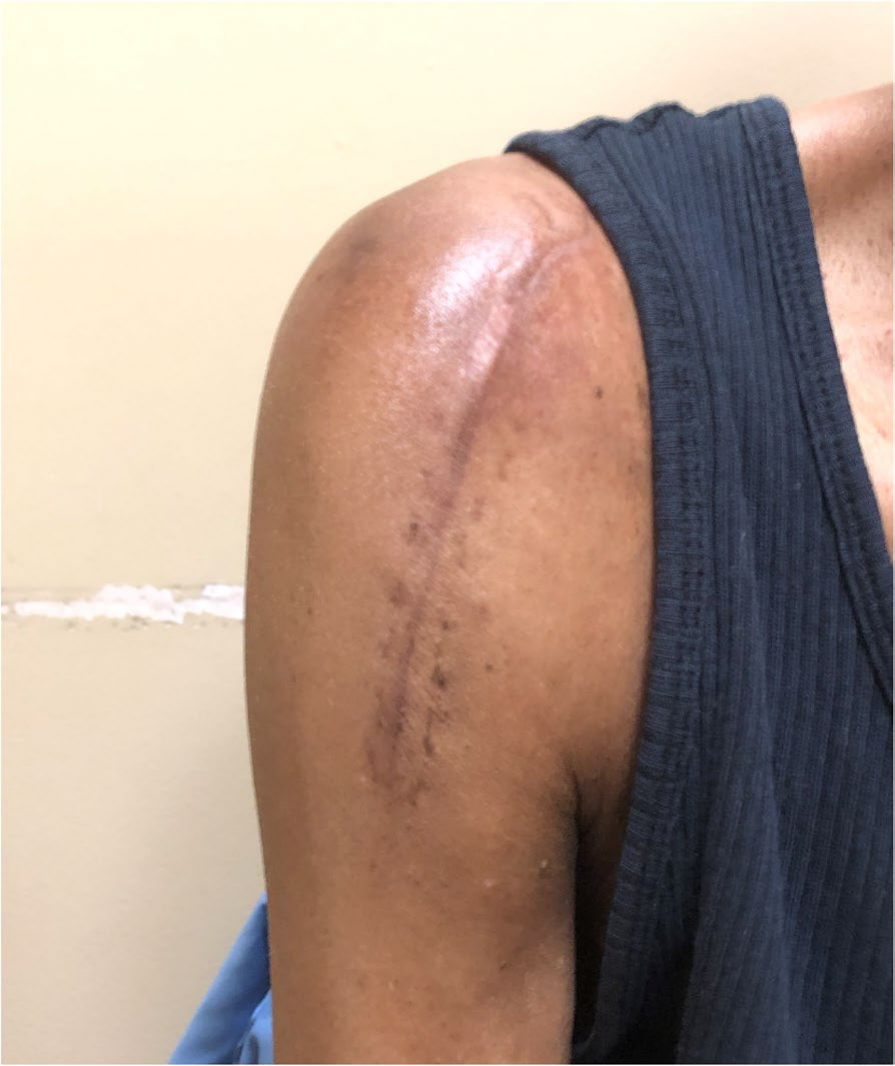
Clinical photograph of the right shoulder at 1 year from the first stage demonstrating resolution of the cyst and appropriate contour of the shoulder

The patient followed up again two weeks later with complaint of recurrence of the swelling, this time with a newly formed draining fistula (Fig. 4). Given the persistence of the swelling and newly formed drainage sinus, the patient was placed on prophylactic antibiotics in the form of cephalexin 250 mg every 6 h orally and referred for surgical evaluation. At the time of orthopedic consultation, the patient’s fistula had persistent drainage for nearly one month despite oral antibiotics and pressure dressings. Laboratory evaluation demonstrated an elevated sedimentation rate of 41 mm/hr (ref range 0–30 mm/hr), with a C-Reactive Protein level within normal limits. Given the continuous draining fistula communicating with the intra-articular joint due to the massive retracted irreparable RC tear as well as elevated inflammatory marker in a diabetic patient, the concern for infection was high. Due to the patient’s end stage RC-tear arthropathy in the setting of a failed prior RC repair, the ultimate treatment option for the shoulder would be a reverse total shoulder arthroplasty. As such, the concern for periprosthetic infection is high. If the patient developed a postoperative periprosthetic infection, she would require a two-stage revision with inferior outcomes. Thus, the proposed surgery was a staged procedure with the first stage including excision of the cyst, resection hemiarthroplasty, placement of an articulating antibiotic spacer, and deltoid and trapezial muscle mobilization to cover the draining sinus (Fig. 5). The antibiotic spacer was hand molded and contained 2 g of Tobramycin and 2 g of Vancomycin. During the initial stage, multiple culture specimens were taken and held for 14 days and reveal no growth, however, the patient had been on oral antibiotics at that point. Pathology specimen at that time reviewed fibroadipose and fibromuscular tissue with mild chronic inflammation. The patient was continued on the oral antibiotic regimen above for 21 days following the initial stage. The wound was completely healed, there was no drainage, and no recurrence of the cyst due to the muscle mobilization. Given the negative cultures and absence of signs of infection, the antibiotics were discontinued, and a second stage was recommended. At this time, however, the patient was not interested in a repeat procedure as she reported minimal pain and due to the peak of the recent COVID-19 pandemic. At six months post-operatively, the patient desired improved motion as she only had 95 degrees of forward elevation and was thus indicated for a second stage conversion to a reverse total shoulder arthroplasty. Repeat inflammatory markers at this time were both within normal limits. The second stage involved removal of the antibiotic spacer and implantation of a reverse total shoulder arthroplasty (Fig. 6). At one-year follow-up, the patient has near full range of motion, visual analog score for pain of 1/10, no evidence of infection, and no recurrence of the acromioclavicular joint cyst (Fig. 7).

## Discussion and conclusions

An AC joint cyst or pseudotumor is a relatively rare condition. There are two distinct etiologies of AC cysts. Type 1 is isolated to the AC joint and is the result of AC joint degeneration with subsequent increased fluid production [1]. Type 2, similar to this case, is related to a tear or rupture of the RC. This may result in instability of the GH joint, proximal migration of the humeral head, and deterioration of the inferior capsule of the AC joint. Excess synovial fluid resulting from disruption of the GH joint subsequently migrates through the ruptured RC to the AC joint [2, 3]. The resulting cystic swelling overlying and adjacent to the AC joint is also known as the “Geyser” sign on Magnetic resonance or ultrasound imaging [6].

Signs and symptoms can be variable, depending on the time of presentation. As the disease is usually a rare result of advanced AC joint arthritis or RC arthropathy, patients present with signs and symptoms of RC impingement and tear. Some patients may also complain of generalized discomfort or be completely asymptomatic [6]. Cosmetically, patients may complain of a “lump” or disfigurement in the area of their shoulder. The lump itself is typically non-tender, non-mobile, and firm [7]. The patient may also report some restriction in moving their arm due the lump [7, 8].

Conservative management including observation may be used initially in asymptomatic individuals who are also not concerned with cosmesis. Aspiration and steroid injection of the cyst has been reported as one method of non-surgical management of these lesions, however, there is a high rate of recurrence [5, 9]. Gumina et al. retrospectively observed four patients with type 2 AC joint cysts who underwent cyst aspiration and injection of steroid; all four patients had cyst recurrence within two weeks after the procedure. The authors reported no change in range of motions, visual analog scale, and Constant scores in all patients at one month following the procedure and felt that aspiration of these cysts is a useless practice [5]. It is worth to note that spontaneous resolution of these cysts has been reported in only two cases in the literature with observation only and no other medical intervention [2, 10].

Surgical treatment for type 1 AC joint cysts which do not communicate with the glenohumeral joint include cyst excision, distal clavicle resection, and open AC joint irrigation and debridement [1]. Severe different surgical operative techniques for management of type 2 AC joint cysts were reported in the literature; including total shoulder arthroplasty in patients with a repairable rotator cuff, and hemiarthroplasty [10, 11]. Reverse total shoulder arthroplasty has also been reported in only two cases of large AC joint cysts in the setting of massive RC-tear arthropathy [11, 12].

A review by Christodoulou et al. of 57 AC joint cyst cases revealed that type 2 cysts are more common that type 1, and both types had better outcomes managed surgically than conservatively; however, most reported cases lacked long-term follow-up to better evaluate both methods of treatment [4].

Our patient had a continuous draining AC joint cyst fistula following aspiration and steroid injection. The concern for high risk of infection in the setting of the patient’s history of diabetes mellitus and draining cyst, that would necessitate a two-stage revision. As a result, the decision was made to proceed with a staged reverse shoulder arthroplasty given the setting of a massive irreparable rotator cuff tear and end stage cuff tear arthropathy. This novel approach should be considered in the setting of an immunocompromised patient when there was a concern for periprosthetic infection.

We report a case of an AC joint cyst in a diabetic patient with RC-tear arthropathy complicated by a draining fistula as a result of cyst aspiration and steroid injection. Although aspiration of cysts has been suggested as a treatment option, we do not recommend this intervention as it has a high rate of recurrence and may result in draining fistula formation. A trial of conservative management (with physical therapy and observation) may be considered if surgery is not contemplated, as there is some literature to support trying conservative management first.

In the case we present, due to the persistent drainage of the cyst following aspiration and steroid injection, and the concern for infection, the patient was treated with a staged reverse shoulder arthroplasty given the setting of an irreparable RC tear and end stage RC-tear arthropathy. As such, the concern for periprosthetic infection is high. If the patient developed a postoperative periprosthetic infection, she would require a two-stage revision with inferior outcomes. Thus, the proposed surgery was a staged procedure.

## Data Availability

Not applicable.
